# 148 The energy expenditure of different strategies to break up prolonged sedentary behaviour in office workers

**DOI:** 10.1093/eurpub/ckae114.189

**Published:** 2024-09-26

**Authors:** Jen Vanherle, Ine Nieste, Wouter Franssen, Bert Op ‘t Eijnde

**Affiliations:** SMRC Sports Medicine Research Center, BIOMED, Biomedical Research Institute, Faculty of Health and Life Sciences, Hasselt University, Hasselt, Belgium; SMRC Sports Medicine Research Center, BIOMED, Biomedical Research Institute, Faculty of Health and Life Sciences, Hasselt University, Hasselt, Belgium; Department of Nutrition and Movement Sciences, Nutrim, School of Nutrition and Translation Research Maastricht, Faculty of Health, Medicine and Life Sciences, Maastricht University, Maastricht, The Netherlands; SMRC Sports Medicine Research Center, BIOMED, Biomedical Research Institute, Faculty of Health and Life Sciences, Hasselt University, Hasselt, Belgium; SMRC Sports Medicine Research Center, BIOMED, Biomedical Research Institute, Faculty of Health and Life Sciences, Hasselt University, Hasselt, Belgium; Division of Sport Science, Faculty of Medicine and Health Sciences, Stellenbosch University, Stellenbosch, South Africa

## Abstract

**Background:**

Since the millennium shift, the number of people who work in an office environment has increased up to 30%. This leads to an increase in sedentary behaviour (SB), which has been associated with numerous chronic diseases such as type 2 diabetes mellitus and cardiovascular disease. The underlying mechanisms are attributed to metabolic processes, including a higher postprandial glucose and lipid concentration, associated with the low energy expenditure of SB.

**Purpose:**

As studies now show that interrupting SB can acutely improve metabolic markers such as glucose regulation, this is an emerging field of research. Even more so because interruptions in SB, in terms of intensity, range from standing breaks to high-intensity physical activity breaks. In order to better compare different strategies for interrupting SB in an office environment, it is important to know the energy expenditure associated with these strategies. Therefore, this study was performed to compare the energy expenditure of two different strategies to interrupt prolonged SB that can be implemented in an office environment to enhance cardiometabolic health.

**Methods:**

In this cross-sectional observational study, indirect calorimetry was used to determine the energy expenditure in three different simulated office environments (prolonged SB, 2min walking breaks @ 3km/h, standing breaks) for a duration of 15 minutes. This technique is based on measuring the oxygen uptake and CO2 production to calculate the associated energy expenditure and substrate oxidation.

**Results:**

Eleven participants completed all simulated office environments. There was a significant increase in energy expenditure in standing breaks compared to prolonged SB (+0.11 kcal/min, p = 0.02) and walking breaks compared to both prolonged SB (+0.49 kcal/min, p < 0.001) and standing breaks (+0.38 kcal/min, p < 0.001).

**Conclusions:**

This study suggests that indirect calorimetry can be used to determine the energy expenditure of different strategies to interrupt prolonged sedentary behaviour. Future studies should consider energy expenditure when studying the cardiometabolic health effects of reducing sedentary behaviour by implementing interruptions.

**Support/Funding Source:**

This study was funded by Hasselt University.

